# Development of Ethosomes for the Topical Treatment of Androgenic Alopecia: Ethanol Effect on Dutasteride Targeting to the Hair Follicles

**DOI:** 10.3390/pharmaceutics17060786

**Published:** 2025-06-17

**Authors:** Jayanaraian F. M. Andrade, Rafael V. Rocho, Breno N. Matos, Geisa N. Barbalho, Kariane M. Nunes, Marcilio Cunha-Filho, Guilherme M. Gelfuso, Tais Gratieri

**Affiliations:** 1 Laboratory of Food, Drugs, and Cosmetics (LTMAC), University of Brasilia (UnB), Brasília 70910-900, DF, Brazil; mjayanaraian@gmail.com (J.F.M.A.); rafael.rocho@aluno.unb.br (R.V.R.); brenomatos15@hotmail.com (B.N.M.); geisabarbalho@gmail.com (G.N.B.); marciliofarm@hotmail.com (M.C.-F.); 2 Pharmaceutical and Cosmetic Research and Development Laboratory, Institute of Public Health, Federal University of Western Pará (UFOPA), Santarem 68040-255, PA, Brazil; kariane.nunes@gmail.com

**Keywords:** androgenic alopecia, dutasteride, follicular targeting, ethosomes

## Abstract

**Background/Objectives:** Treatment options for androgenic alopecia are still very limited and lack long-term efficacy. Dutasteride (DUT) has gained interest as a potent inhibitor of 5α-reductase, allowing for spaced applications, but DUT oral intake can cause serious adverse effects. Herein, we developed, characterized, and assessed the potential of DUT-loaded ethosomes with increasing ethanolic concentrations for hair follicle (HF) targeting to treat androgenic alopecia, hypothesizing that ethanol’s interaction with HFs’ sebum might increase DUT targeting to the HFs. **Methods:** Ethosomes were obtained using the water-dropping method. After a hydrodynamic size screening, a 30% ethanol concentration was fixed. Ethosomes with 30% ethanol were also prepared and had their ethanolic content removed by rotary evaporation for the evaluation of ethanol in targeting DUT to the HFs. The targeting factor (Tf) was calculated as the ratio between the DUT amount in HFs and the total DUT amount recovered from all skin layers after in vitro porcine skin penetration tests for 12 and 24 h. **Results:** The ethanolic concentration affected the vesicles’ size and the targeting potential. While the dried ethosomes could not increase DUT accumulation in the HFs at both time points (Tf: 0.27 in 12 h and Tf: 0.28 in 24 h), the presence of 30% ethanol in the vesicles increased the Tf from 0.28 (12 h) to 0.34 (24 h), significantly superior (*p* < 0.05) than the dried ethosome and control (Tf: 0.24) in 24 h. **Conclusion:** Ethosomes with a 30% ethanolic concentration were slightly more efficient in targeting HFs for dutasteride delivery.

## 1. Introduction

Androgenetic alopecia (AGA), also known as male or female pattern hair loss, affects a significant portion of the adult population and is primarily driven by genetic predisposition and androgenic influences [1,2,3]. The progression of AGA is largely attributed to the accumulation and action of dihydrotestosterone (DHT), a potent androgen formed through the conversion of testosterone by the enzyme 5α-reductase [4,5]. This enzyme can be found in two primary isoforms, type I and type II, which are differentially distributed in the scalp and hair follicles (HFs) [6,7,8].

Currently, Food and Drug Administration (FDA)-approved treatments for AGA consist of oral finasteride and topical minoxidil. Minoxidil, a vasodilator with hair growth-promoting effects [9,10], has its efficacy limited by physicochemical properties, namely low aqueous solubility, which leads to irritation and dryness caused by its alcohol-based formulations [11,12,13]. Meanwhile, finasteride, a selective type II 5-reductase inhibitor, reduces DHT levels by 70%, slows hair loss, and promotes hair regrowth [14,15,16]. However, its oral administration is linked to adverse sexual side effects, like decreased libido, ejaculatory disorders, and erectile dysfunction, which limits its acceptance and adherence among patients [17,18,19,20]. There is also an increasing concern about its long-term psychological effects on patients [18,21,22].

In the same class and more recently, dutasteride (DUT) has emerged as a promising alternative to finasteride for AGA. Unlike finasteride, DUT is a dual inhibitor of both 5α-reductase isoforms, making it a more potent DHT suppressant [23,24]. This potency allows for extended intervals between smaller doses, which is beneficial for treatment adherence. However, when administered orally, DUT may cause similar sexual and psychological side effects to those observed with finasteride, posing a significant drawback [25,26]. Herein, the ideal scenario would be a topical administration of DUT in a manner able to target the drug to the HFs while limiting the absorption by subjacent tissues, potentially mitigating the adverse sexual effects associated with oral administration.

Notwithstanding, DUT’s high lipophilic character (log *p* = 6.8) challenges its topical target delivery. This might be the reason why DUT has been little explored in drug delivery studies for AGA compared to other drugs such as minoxidil and finasteride. In the opportunities where it was successfully loaded into nanocarriers, many studies failed to verify whether or not DUT was directed to the HFs [27], an aspect of utmost importance to reduce the chances of causing the cited unwanted adverse effects, also making it difficult to compare the follicular targeting potential of different nanocarriers.

The follicular targeting factor (Tf) shows how much of a topically applied formulation is in HFs at one specific time point. For that, the penetrated amount of the interest drug must be quantified in all skin layers, i.e., the stratum corneum, HFs, and viable skin. The Tf is the ratio between the penetrated amount in HFs compared to the penetrated drug amount in all skin layers. Therefore, the Tf is an index that ranges between 0 and 1, with 0 meaning no drug was quantified in HFs and 1 meaning all the administered drug was in HFs. For a drug with a high potential to cause side effects like DUT, a Tf closer to 1 is highly desirable, as the topically applied formulation would be concentrated in HFs for a local effect rather than in the other layers. From the studies that investigated the targeting potential of nanocarriers to the HFs, four particles stand out.

Firstly, a poly(lactic-co-glycolic) acid (PLGA) nanoparticle coated with HF derma papilla cells was produced to increase biocompatibility. During in vitro studies in porcine ear skin for 12 h, the particles targeted 41% (Tf = 0.41) of the applied dose to the HFs [28]. However, the intricate obtainment process of such particles may limit their clinical use. Secondly, a similar targeting effect was observed by our research group with lipidic-core PLGA nanocapsules coated with chitosan, which delivered 40% (Tf = 0.40) of the DUT dose to the HFs of porcine ear skin during in vitro tests after only 6 h when a 3 min massage followed the application [29]. Thirdly, besides the positive results, we aimed for a higher targeting effect, so the group also developed iron oxide nanocarriers able to target 51% (Tf = 0.51) of the DUT dose in 24 h to the HFs of porcine ear skin in vitro. Nonetheless, the iron presence in this formulation leaves a dark coloration on the skin after application, which limits its use to dark-haired patients [30].

Fourthly and more recently, searching for a more patient-friendly option, we also obtained and evaluated the targeting potential of DUT-loaded liposomes with different bilayer structures [31]. The best performance was achieved with ultra-deformable liposomes, also known as transfersomes, which targeted 32% (Tf = 0.32) of the DUT delivery to the HFs in 12 h, decreasing to 27% (Tf = 0.27) in 24 h in vitro skin penetration tests in porcine ear skin. In this context, aiming to increase DUT targeting to the HFs, we now propose DUT loading in ethosomes.

Ethosomes are lipid-based nanocarriers composed of phospholipids, ethanol, and water, which can load lipophilic drugs such as DUT. Their flexible structure allows them to penetrate the deeper layers of the skin more effectively than traditional lipid-based systems, providing a non-invasive delivery route [32]. Additionally, the ethanol interaction with HFs’ sebum might influence the target potential of the formulation. Moreover, the ethanolic content in ethosomes can be optimized to minimize skin irritation while maintaining effective drug delivery, offering a more tolerable formulation than standard alcoholic vehicles used in minoxidil. Therefore, this work aimed to obtain DUT-loaded ethosomes with increasing amounts of ethanol up to 45% and evaluate the potential of such a drug delivery system to target HFs, while also assessing the ethanol influence in the targeting process, for more effective androgenic alopecia treatment.

## 2. Materials and Methods

### 2.1. Materials

DUT was acquired from Henan Tianfu Chemical Co. (Zhengzhou, China). Phosphatidylcholine (PC) from soybean was bought from Lipoid (S 100-PC ≥ 94.0%, Ludwigshafen, Germany). Sodium acetate was acquired from Sigma-Aldrich (St. Louis, MO, USA). Ethanol and sodium dodecyl sulfate (SDS) were obtained from Dinâmica (São Paulo, Brazil). Acetonitrile was bought from J. T. Baker (New Jersey, NJ, USA). Scotch n^o^. 845 Book Tape was acquired from 3 M (St. Paul, MN, USA). Cyanoacrylate glue was acquired from Henkel Loctite (Dublin, Ireland). Water was purified by a Milli-Q system (Millipore, Burlington, MA, USA) with a 0.22 µm pore size end filter. All other chemicals and reagents were of analytical grade. Dialysis tubing cellulose membranes (D9652-100 FT) were purchased from Sigma-Aldrich (St. Louis, MO, USA).

The porcine ears were obtained from a local slaughterhouse (Suino Bom Alimentos LTDA., Brasilia, Brazil) shortly after animal sacrifice from animals intended for human consumption, according to the main ethical guidelines and regulatory standards [33,34,35]. Skin samples extracted from the ears and cleaned were stored at −20 °C for a maximum of 1 month before use.

### 2.2. Ethosome Preparation

Ethosomes were prepared using the water-dropping technique [36]. Briefly, PC (200 mM) and DUT (17 mM) stock solutions were prepared in ethanol. The DUT (17 mM) solution volume was fixed at 0.30 mL to reach the target concentration of 0.30 mg/mL. PC (200 mM) solution volume varied between 0.7 and 4.2 mL to form ethosomes with ethanolic concentrations between 10% and 45%. Purified water was dropped with a syringe into the ethanolic solution containing PC and DUT until a final volume of 10 mL at 30 °C under constant stirring at 750 rpm. The ethosomes were kept under stirring for 30 min after water addition. The ethosomes’ compositions are listed in Table 1.

After a size screening, ethosomes with 30% ethanol content were selected to continue the experiments. ET30 was concentrated in a rotary evaporator (IKA Lab Dancer, IKA, Staufen, Germany) at 30 °C to 7 mL for ethanol removal. The volume was subsequently increased to 10 mL with purified water. The ethosomes that underwent this process were named ET30-EF (ethanol-free). The final pH of the formulations was adjusted to 7.4.

### 2.3. Characterization of DUT-Loaded Ethosomes

#### 2.3.1. Ethosomes’ Size and Zeta Potential

The hydrodynamic size and polydispersity index (PdI) were evaluated by dynamic light scattering, while the zeta potential was measured through electrophoretic mobility (Zetasizer Nanoseries, Malvern Instruments, Worcestershire, UK) using the samples diluted in purified water (1:100 *v*/*v*) at 25 °C. All measurements were performed in triplicate.

#### 2.3.2. Determination of DUT Content

For total DUT quantification in the control and formulations (ET30 and ET30-EF), 100 µL of the formulations was diluted in methanol (1:10 *v*/*v*) and vortexed for 60 s. Then, the samples were filtered with a 0.45 µm syringe filter into a vial and stored for posterior analysis.

#### 2.3.3. Morphological Analyses

Morphological analyses were accomplished with a transmission electron microscope (TEM; JEM 1011 Transmission Electron Microscope, JEOL, Tokyo, Japan—100 kV). Micrographs were taken with a GATAN BioScan camera (model 820, GATAN, Pleasanton, CA, USA) using Digital Micrograph 3.6.5 software (GATAN, Pleasanton, CA, USA). For the images, diluted aliquots of ethosomes were deposited on a Formvar-coated copper grid (Electron Microscopy Sciences, Pleasanton, CA, USA) and left to dry for 10 min. The excess of formulations was absorbed with a piece of filter paper. Following, a 3% uranyl acetate solution (*w*/*v*) was added and also left to dry for 10 min. The excess was also removed with the aid of filter paper.

#### 2.3.4. Viscosity

The viscosity of the ethosomal formulations was evaluated using a rotational rheometer (HR-2 Discovery, TA Instruments, New Castle, DE, USA) equipped with a 1° cone-plate geometry and a 50 mm diameter plate. The gap between the plates was set to 25 mm. Each sample was placed onto the Peltier plate and equilibrated at 25 °C for 30 s before measurement. Flow behavior was assessed by performing a controlled shear rate test consisting of an ascending ramp from 0.1 to 10 s^−1^ over 60 s, followed by a descending ramp from 10 to 0.1 s^−1^ over an additional 60 s [37].

### 2.4. Drug Release

In vitro drug release was determined using a cellulose membrane (14 kDa) mounted on a Phoenix DB-6 diffusion cell assembly (Teledyne Hanson Research Inc., San Francisco, CA, USA) (diffusional area = 1.77 cm^2^). The tested ethosomes and the control contained 0.30 mg/mL of DUT and were evaluated in quintuplicate. In total, 500 µL of each formulation was placed in the donor compartment. The receptor compartment contained 15 mL of 59.5% water:0.5% Tween 80:40% ethylene glycol solution to ensure sink conditions, as previously described [29]. The system was kept under magnetic stirring (300 rpm). Samples of 1 mL were removed from the receptor solution every hour for 12 h and then at 24, 36, and 48 h for DUT quantification. To maintain the same conditions, the same volume of fresh receptor solution was replaced in the receptor compartment. A DUT oil solution was used as a control.

The Excel add-in DDSolver version 1.0 was used to analyze the drug release kinetics of DUT from the ethosomal formulations [38]. This software applies nonlinear optimization techniques to fit release data to multiple kinetic models, including zero-order, first-order, Higuchi, and Korsmeyer–Peppas models. The most suitable model was selected based on statistical criteria such as the adjusted coefficient of determination (R^2^ adjusted) and the Model Selection Criterion (MSC). To further elucidate the release mechanism, the Korsmeyer–Peppas model was applied to calculate the release exponent “n”, which characterizes the release behavior as Fickian diffusion, anomalous transport, or case II transport [39].

### 2.5. In Vitro Skin Penetration Tests

The in vitro skin penetration tests were conducted with full-thickness porcine ear skin mounted in a Phoenix DB-6 diffusion cell assembly (Teledyne Hanson Research Inc., San Francisco, CA, USA) (diffusional area = 1.77 cm^2^). For each tested sample, 500 µL was added to the donor chamber. The formulations remained in contact with the stratum corneum for 12 or 24 h, while the receptor chamber was filled with 15 mL of 0.5% SDS aqueous solution [29]. DUT was extracted from the skin layers at the end of each experiment following the differential stripping method [40]. Briefly, at the end of the experiments, the skin was rinsed with purified water to remove the rest of the formulation and gently dried with a paper towel. Then, each skin sample was placed on a flat surface with the stratum corneum facing upwards. The stratum corneum was removed by applying and removing 15 adhesive tapes. Following, HFs were removed by putting a drop of cyanoacrylate glue in the permeated area and covering it with one more adhesive tape. The tape was removed after total glue polymerization (±2 min). This process was repeated to guarantee complete HF removal. Finally, the remaining skin was fragmented into pieces with scissors. DUT extraction was performed by adding 2.5 mL of methanol to tubes containing the skin layers, which were kept under rotation in a multi-rotator (model Multi Bio RS-24, BioSan, Riga, Latvia) for 24 h. DUT recovery from the stratum corneum, HFs, and viable skin (viable epidermis and dermis) was previously determined with values higher than 97% [29].

The follicular targeting effect (T_f_) was calculated following Equation:

$$T_{f}=\frac{{DUT}_{HF}}{\sum DUTpenetrated}$$

where “DUT_HF_” is the DUT amount extracted from the HFs, and “∑DUT_penetrated_” is the total DUT amount recovered from stratum corneum, HFs, and viable skin.

### 2.6. Analytical Method

DUT quantification was performed by High-Pressure Liquid Chromatography (HPLC; model LC-20 CE, Shimadzu, Japan) with UV detection (SPD-M20A) at 242 nm. The mobile phase was purified water and acetonitrile (47:53 *v*/*v*) in the isocratic mode. The flow rate was 1 mL/min, and the oven temperature was 40 °C. An RP Luna C8(2) column (150 × 4.6 mm; 5.0 μm, Phenomenex^®^) was used as the stationary phase. The injection volume of samples was 20 μL. This method was previously validated by our group following the International Conference on Harmonization (ICH) guidelines [29]. It was considered selective and linear in the concentration range of 0.25–120.0 µg/mL (r = 0.9999; y = 22,858x + 3635.2). The intra- and inter-day precision and accuracy of the method showed a coefficient variation (CV%) and relative error (E%) < 5%. The limit of quantification (LOQ) and limit of detection (LOD) were 0.41 and 0.14 µg/mL, respectively.

### 2.7. Statistical Analysis

The statistical significance of the data was evaluated by analysis of the variance (ANOVA) with Tukey’s post hoc test. The significance level was fixed at 0.05. All data were expressed as the mean ± standard deviation.

## 3. Results and Discussion

### 3.1. Ethosome Characterization

**Figure 1:**
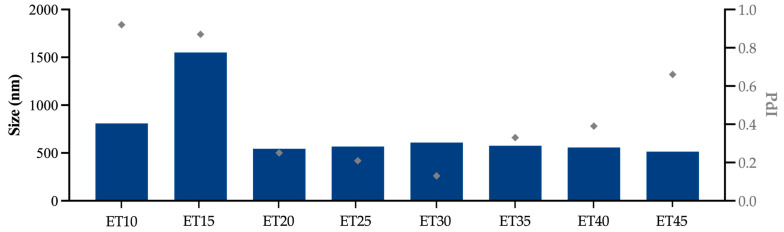
Influence of the ethanol concentration on the hydrodynamic size and PdI of ethosomes. F: filtered samples.

Ethosomes with eight different compositions were produced with increasing ethanol concentrations from 10% to 45%. The PC content also increased with the total ethanolic content (Figure 1). Higher concentrations of ethanol during the ethosome obtainment process are related to reducing the vesicle size and the PdI [41]. In this study, the ethanol increase led to a reduction in vesicle sizes from ET15 to ET45, except for ET30, which was slightly bigger than ET25, although this difference was not significant (*p* > 0.99). Regarding the PdI, the ethanol increase caused a reduction in every ethosome PdI from ET10 to ET30, and then it started to increase again.

Ethosomes with 20% ethanol content and above achieved the size of interest, around 600 nm, reaching the highest value with 30% ethanol (ET30) content, 608.4 ± 43.3 nm, with the lowest PdI (0.13 ± 0.09). Therefore, these ethosomes were selected for further experiments. The size of interest was chosen following a classic study in the field, which discovered that nanoparticles with sizes around 650 nm could reach deeper in HFs, where DUT targets are found, compared to larger and smaller ones [42]. Even though other recent studies corroborate these findings [43,44], there is no consensus in the literature on the ideal size to target hair follicles [45]. Some authors suggest HF targeting is better at 300 nm [46], while others found 200 nm and smaller to be more suitable [47,48]. Such different results might indicate that more factors than just the nanocarrier size are involved in the process of targeting HFs.

**Figure 2:**
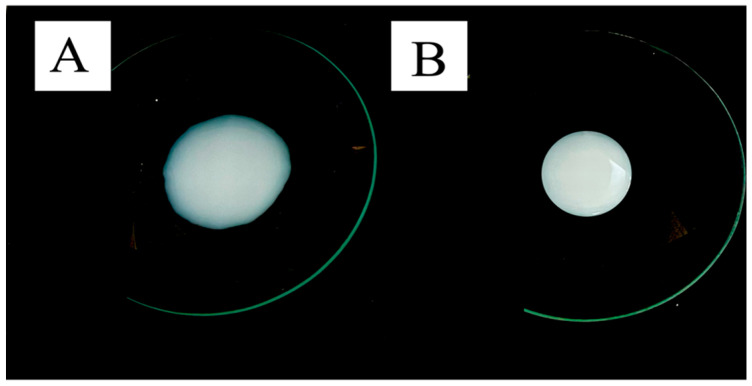
Visual aspect of ethosomes. (**A**) ET30 and (**B**) ET30—EF.

To verify ethanol’s influence on targeting HFs, ET30 was also prepared and had the ethanol removed, in which case it was named ET30—EF (ethanol-free). Visually, both ethosomes appeared as a whitish liquid, but ET30—EF was slightly more viscous than ET30 (Figure 2). ET30’s measured viscosity was 0.0425 ± 0.01 Pa·s, while ET30—EF’s viscosity was 0.151 ± 0.01 Pa·s. 

**Figure 3:**
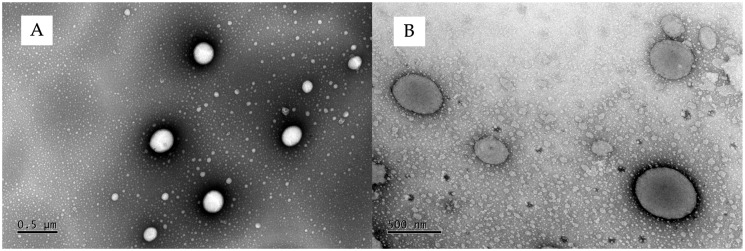
TEM micrographs of DUT-loaded ethosomes. (**A**) ET30, 8Kv, at 6000×; (**B**) ET30-EF, 8Kv, at 10,000×.

Although the drying process caused a reduction in the hydrodynamic size of the vesicles (ET30—EF), it did not significantly alter their size (*p* > 0.05) (Table 2). The transmission electron microscopy also showed that the ethanol removal drying process did not affect the ethosomes’ shape, showing oval vesicles for the ET30-EF samples (Figure 3B), like the ones from the ET30 samples (Figure 3A).

### 3.2. In Vitro Drug Release

**Figure 4:**
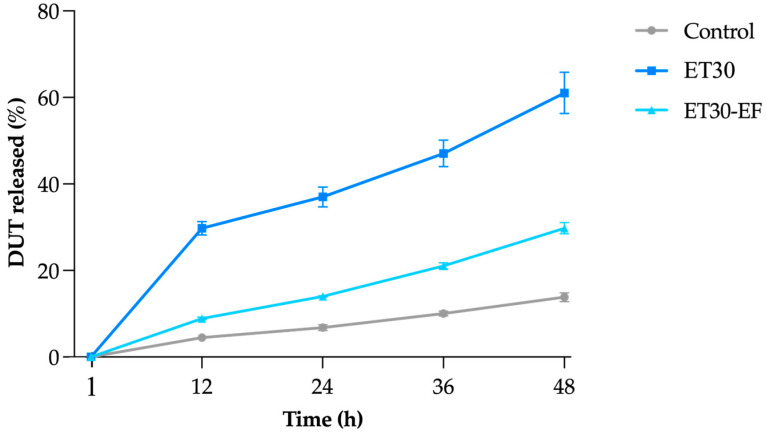
Release of DUT from ET30 ethosomes, ET30-EF, and control (oil solution of DUT) over 48 h. Data expressed as mean ± SD (*n* = 5).

Figure 4 shows the percentage of DUT released from ET30, ET30-EF, and the control, an oily solution of DUT. It is worth noting that an oily formulation of DUT would not be viable in a clinical case, since its application would be unpleasant for the patient, leaving the hair with a greasy appearance. Other options, such as an ethanolic solution with 30% ethanol content, would be more suitable for the control; however, the solubility of DUT in this medium (1.73 ± 0.25 µg/mL) was insufficient to achieve the same DUT concentration in the ethosomes, around 0.30 mg/mL; therefore, we decided to use DUT in mineral oil, a medium where the drug is completely solubilized.

The formulations as well as the control demonstrated a slow and prolonged release profile; the incomplete release observed over 48 h is consistent with the known characteristics of such vesicular delivery systems, which typically exhibit retentive behavior and modulated diffusion of hydrophobic drugs (Figure 4) [49]. Both formulations released more DUT than the control (*p* < 0.05), which was expected, considering DUT, as lipophilic as it is (log *p* = 6.8), has more affinity with mineral oil and tends to diffuse slower to the more hydrophilic medium in the acceptor compartment. ET30 released significantly more DUT than the dried control ethosome at all time points (*p* < 0.05), i.e., 61.0 ± 4.8% of DUT in 48 h compared to 29.8 ± 1.3 and 13.8 ± 1.0% released from the dried ethosome and control, respectively. To explain the higher release of DUT from ET30, it was hypothesized that part of the ethanol was solubilized in the water, carrying with it a portion of the DUT that was solubilized in ethanol. In fact, it has already been reported in the literature that ethanol can accelerate the release of drugs [50].

The differences in drug release profiles between ET30, ET30-EF, and the control formulation can be partially attributed to their distinct physical characteristics. Notably, ET30 exhibited a significantly lower viscosity (0.0425 ± 0.01 Pa·s) compared to ET30-EF (0.1511 ± 0.01 Pa·s). This lower viscosity results in a more fluid system, which may facilitate drug diffusion. In contrast, the increased viscosity of ET30-EF implies a denser matrix, which can hinder the diffusion of DUT and decrease its release rate. This correlation between a lower viscosity and enhanced drug release has been previously reported for vesicular and semisolid systems in topical delivery, where the viscosity directly influences the mobility of drug molecules and their ability to diffuse through the matrix [51]. Furthermore, the control formulation, composed of DUT dissolved in mineral oil, lacks both a vesicular structure and an aqueous phase, limiting drug partitioning and diffusion into the receptor medium.

The release profiles of the formulations were thoroughly analyzed using various mathematical models, including zero-order, first-order, Higuchi, and Korsmeyer–Peppas models. Table 3 presents the values obtained for each evaluated kinetic parameter. Notably, the first-order model demonstrated the strongest correlation with the release profile for ET30. According to this model, the amount of drug released is proportional to the remaining quantity within the vesicles over time, gradually decreasing [52]. This finding supports the significantly higher release rate of DUT observed for ET30 within the first 12 h (*p* < 0.05) when compared to ET30-EF and the control. The highest MSC and R^2^-adjusted values for ET30-EF and the control group were associated with the Korsmeyer–Peppas model, which is commonly applied to systems governed by more than one release mechanism, such as drug diffusion and matrix relaxation [39,53].

ANOVA, followed by Tukey’s test, was applied to assess the statistical significance between the kinetic models exhibited by the formulations and the control. Only the formulation ET30-EF showed a similarity between the zero-order and Korsmeyer–Peppas models, with no significant difference (*p* > 0.05). This finding supports the low release rate of dutasteride observed in the release profile, which can be attributed to the absence of ethanol in the formulation combined with the drug’s high lipophilicity, given that the sink condition was maintained.

The Korsmeyer–Peppas equation was also used to determine the mechanism of drug release from ethosomes by the release diffusional exponent “*n*”. Supposing the n-value is >0.45 or <0.89, the mechanism follows non-Fickian (anomalous) diffusion. When n = 0.89, it will be non-Fickian case II; if n > 0.89, it will be non-Fickian super case II transport [53]. The value of *n* > 1 found for both formulations and the control, which follows the non-Fickian super case II model, suggests that the release rate is controlled by more than one mechanism, including drug diffusion through the solvent and relaxation caused by destabilization of the lipid bilayer matrix of the ethosomes [54].

### 3.3. In Vitro Skin Penetration Experiment and Follicular Targeting Effect

**Figure 5:**
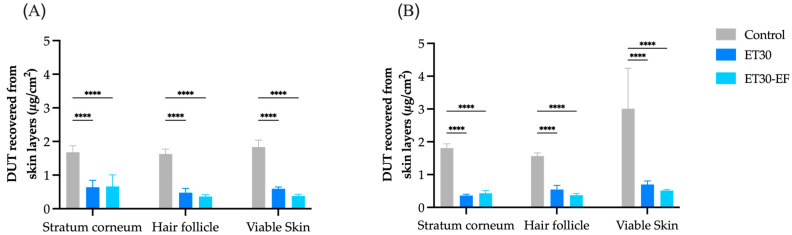
DUT amounts recovered from stratum corneum, hair follicles, and viable skin after 12 h (**A**) and 24 h (**B**) during skin penetration test in vitro with ET30, ET30-EF, and control. Data are expressed as mean ± SD (*n* = 6). **** *p* < 0.0001.

**Figure 6:**
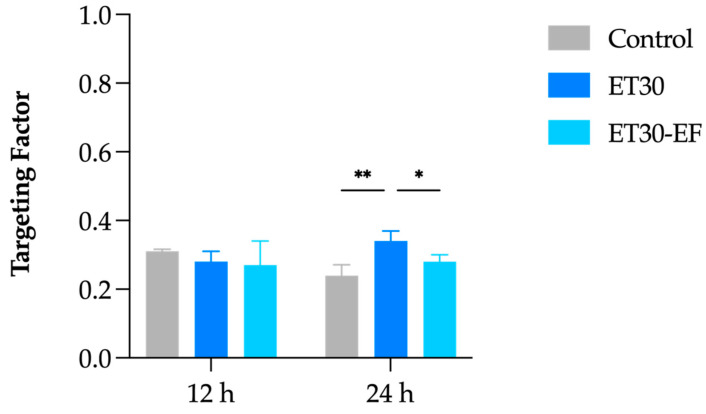
Follicular targeting factor of DUT-loaded ethosomes ET30, ET30-EF, and control after 12 and 24 h of in vitro skin penetration tests. ** *p* < 0.003 and * *p* < 0.03.

The in vitro skin penetration assays were performed at 12 and 24 h (Figure 5). In both cases, the oily control of DUT promoted a greater accumulation of DUT in all skin layers (*p* < 0.05). However, the control did not target DUT to the HFs at either point, as seen in previous work from our group with liposomes, also using DUT in mineral oil as a control [31]. The follicular targeting factor assesses the drug amount accumulated in HFs compared to the other skin layers, i.e., the stratum corneum and viable skin. At 12 h, the calculated targeting factor for control was 0.31, and at 24 h, it decreased to 0.24 (Figure 6), accumulating more DUT in viable skin than in the stratum corneum and HFs.

The skin permeation profile was similar between the two ethosomes (Figure 5), with DUT accumulating in the stratum corneum in 12 h and diffusing to the HFs and viable skin in 24 h in both cases, but without a statistically significant difference between the two formulations (*p* > 0.05). It is important to highlight that the accumulation generated by ethosomes in viable skin was statistically lower than the control (*p* < 0.0001) (Figure 5). Furthermore, DUT was not detected in the receptor compartment (LOD = 0.14 µg/mL) in any case, indicating a lower tendency to be absorbed by nearby tissues and the bloodstream, reducing the risk of causing systemic adverse effects.

Regarding the follicular targeting potential, the targeting effect generated by ET30 increased from 0.28 to 0.34 in 12 h and 24 h, respectively, and it was statistically higher than the targeting factor of the control (0.24 in 24 h) and the dried ethosome (0.28 in 24 h) (*p* < 0.03).

This targeting factor of 0.34 is superior to a different elastic–lipidic vesicle loaded with DUT, in which transfersomes had a targeting factor of 0.32 in 12 h but decreased to 0.27 in 24 h [31]. This difference can be attributed to the presence of ethanol. Indeed, ethanol is a known promoter of transdermal permeation due to its degrading action on the lipids of the stratum corneum [55,56]. It is proposed that ethanol, in addition to dissolving skin lipids, facilitates the entry of solutes into the skin [57,58,59], which may also have occurred in the sebum present in the HFs in the present study. Further studies are needed to confirm this hypothesis.

In addition, as previously discussed, formulations with a high alcohol content (>10%) can cause several adverse effects on the skin, such as dryness, erythema, and contact dermatitis, especially after repeated applications. Although it is a valid concern, various studies assessing the skin irritability of ethosomes with varying ethanolic content have demonstrated that ethosomes are safe for topical use [60,61,62]. For example, ethosomes with a 30% ethanol concentration loaded with caffeic acid had their irritability potential tested by an occlusive patch experiment in twenty healthy human volunteers. The formulation applied in a single dose was left in contact for 48 h, and the skin irritation was evaluated after 15 min and 24 h after patch removal. The formulation was deemed non-irritating [63]. The same protocol with an occlusive patch was used to verify the irritability of dimethyl fumarate-loaded transethosomes [64] and a dimethyl fumarate-loaded ethosome gel [65]; in both cases, the ethosomes had approximately 30% ethanol content, and both were considered not irritating. Furthermore, ethosomes with ethanol concentrations of 20 to 40% were tested on the back skin of rats to assess their long-term tolerability. The animals were treated with 200 µL of the formulation and monitored daily for 10 days. At the end of the test, no skin irritation was identified [41].

Another point that can be considered a limitation of the study is the dose of formulations used in the in vitro tests. A 0.5 mL dose of an aqueous solution would not realistically be in place over 24 h in a clinical scenario. But this setup, called “infinite dose regime”, is more appropriate when the aim is to compare formulations, as it maintains the concentration gradient constant and not varying because of dose depletion caused by evaporation or diffusion into or through the skin [66]. Moreover, the larger volume of the formulation allows for quantifiable drug amounts for the chosen analytical method employed in this study. Indeed, the limit of detection of the methodology is the reason some experiments had to be extended to 24 h, since in 12 h, it was not possible to demonstrate any effects of targeted follicular delivery. However, even after 24 h, the magnitude of the permeation and the actual amount penetrated cannot be extrapolated to the clinical scenario from these experiments, as it only indicates for the best formulation for follicular targeting in vitro.

Furthermore, although the Tf differences are discrete among formulations, it is important to highlight DUT’s potency. The clinical dose of oral DUT is 0.5 mg per day, and oral DUT’s bioavailability in healthy patients is around 60%. The maximum blood concentration after this dose administration is approximately 1.3 ng.mL^−1^ [67]. Hence, finding the best formulation for promoting drug follicular targeting is indeed a promising strategy to determine promising outcomes in vivo.

It is clear that the release profile combined with the potency of the DUT indicates that applications of a formulation containing ET30 would not need to be frequent, and the safety data from the literature about the topical use of ethosomes are encouraging. Nonetheless, further studies are still needed to evaluate the stability, clinical effect, and safety profile of prolonged use of such a formulation.

## 4. Conclusions

Here, DUT-loaded ethosomes were produced with different ethanolic amounts. The results showed that the ethanol concentration affected the vesicles’ size, and it was possible to reach the suitable size to target the HFs of around 500–600 nm with 30% ethanol content. Since ethanolic solutions with higher than 10% alcohol content are potentially skin irritative, the ethanol was removed by rotary evaporation from one sample. The ethanol removal did not significantly affect the morphology of the vesicles or their sizes. However, it affected the release profile and the system’s targeting potential. Ethanol presence caused a quicker DUT release from the ethosomes but also targeted more DUT to the HFs.

In conclusion, this approach indicates towards a more effective treatment for androgenetic alopecia, combining the potent DHT-suppressing effects of DUT with an improved delivery system designed to enhance local efficacy and minimize systemic side effects. Notwithstanding, further studies are warranted to evaluate the clinical efficacy and, mainly, the safety of DUT-loaded ethosomes in AGA therapy.

## Figures and Tables

**Table 1:** Composition variation with increasing ethanol concentrations during the ethosomes obtainment process. The total ethanol amount corresponds to the sum of DUT and PC volumes. Ethosomes (ETs) were named after their ethanolic concentrations (10–45).

Ethosomes	DUT 17 mM	PC 200 mM	Purified Water
ET10	0.30 mL	0.70 mL	9.0 mL
ET15	0.30 mL	1.20 mL	8.5 mL
ET20	0.30 mL	1.70 mL	8.0 mL
ET25	0.30 mL	2.20 mL	7.5 mL
ET30	0.30 mL	2.70 mL	7.0 mL
ET35	0.30 mL	3.20 mL	6.5 mL
ET40	0.30 mL	3.70 mL	6.0 mL
ET45	0.30 mL	4.20 mL	5.5 mL

DUT: dutasteride; PC: phosphatidylcholine.

**Table 2:** Hydrodynamic size, polydispersity index, zeta potential, and drug dose of ET30 ethosomes with 30% alcohol concentration and ET30-EF for ET30 after ethanol removal.

Ethosomes	Hydrodynamic Size (nm)	PdI	Zeta Potential (mV)	DUT Dose (mg/mL)
ET30	604.8 ± 43.3	0.13 ± 0.09	2.2 ± 0.2	0.30 ± 0.01
ET30-EF	581.7 ± 20.5	0.05 ± 0.07	2.7 ± 0.1	0.29 ± 0.01

(*n* = 3). DUT = dutasteride.

**Table 3:** Statistical parameters applied to assess the best fit achieved by applying zero-order, first-order, Higuchi, and Korsmeyer–Peppas models to the DUT release profile.

Samples	Zero Order		First Order		Higuchi		Korsmeyer–Peppas	
	MSC ± SD	R^2^ ± SD	MSC ± SD	R^2^ ± SD	MSC ± SD	R^2^ ± SD	MSC ± SD	R^2^ ± SD
Control	2.03 ± 0.1 ^a^	0.91 ± 0.0 ^a^	1.94 ± 0.1 ^a^	0.90 ± 0.1 ^a^	0.35 ± 0.1 ^a^	0.52 ± 0.0 ^a^	2.41 ± 0.4 ^a^	0.94 ± 0.0 ^a^
ET30	1.88 ± 0.1 ^a^	0.89 ± 0.0 ^a^	2.64 ± 0.2 ^b^	0.95 ± 2.6 ^a^	1.41 ± 0.1 ^b^	0.83 ± 0.0 ^a^	0.84 ± 0.1 ^b^	0.71 ± 0.0 ^a^
ET30-EF	1.92 ± 0.0 ^a^	0.90 ± 0.0 ^a^	1.69 ± 0.0 ^a^	0.88 ± 0.0 ^a^	0.26 ± 0.0 ^a^	0.48 ± 0.0 ^a^	2.25 ± 0.1 ^a^	0.93 ± 0.0 ^a^

Average values sharing the same letter are not significantly different based on the Tukey test (*p* > 0.05).

## Data Availability

Data can be provided by the authors upon reasonable request.
